# Longitudinal image-based prediction of surgical intervention in infants with hydronephrosis using deep learning: Is a single ultrasound enough?

**DOI:** 10.1371/journal.pdig.0000939

**Published:** 2025-08-04

**Authors:** Adree Khondker, Stanley Bryan Z. Hua, Jethro C. C. Kwong, Kunj Sheth, Daniel Alvarez, Kyla N. Velaer, John Weaver, Alice Xiang, Gregory E. Tasian, Armando J. Lorenzo, Anna Goldenberg, Mandy Rickard, Lauren Erdman

**Affiliations:** 1 Department of Surgery, University of Toronto, Toronto, Canada; 2 Division of Urology, Hospital for Sick Children, Toronto, Canada; 3 Department of Computer Science, University of Toronto, Toronto, Canada; 4 Division of Genetics and Genome Biology, Hospital for Sick Children, Toronto, Canada; 5 Center for Computational Medicine, Hospital for Sick Children, Toronto, Canada; 6 Stanford Children’s Health -- Lucile Packard Children’s Hospital, Stanford University, Palo Alto, California, United States of America; 7 Department of Surgery, University of Pennsylvania, Philadelphia, Pennsylvania, United States of America; 8 Division of Urology, Children’s Hospital of Philadelphia, Philadelphia, Pennsylvania, United States of America; 9 Departments of Surgery and Biostatistics, Epidemiology, and Informatics, Perelman School of Medicine, University of Pennsylvania, Philadelphia, Pennsylvania, United States of America; 10 Center for Pediatric Clinical Effectiveness, The Children’s Hospital of Philadelphia, Philadelphia, Pennsylvania, United States of America; 11 Vector Institute, Toronto, Canada; 12 James M. Anderson Center for Health Systems Excellence, Cincinnati Children’s Hospital Medical Center, Cincinnati, Ohio, United States of America; National Yang Ming Chiao Tung University, TAIWAN

## Abstract

The potential of deep learning to predict renal obstruction using kidney ultrasound images has been demonstrated. However, these image-based classifiers have incorporated information using only single-visit ultrasounds. Here, we developed machine learning (ML) models incorporating ultrasounds from multiple clinic visits for hydronephrosis to generate a hydronephrosis severity index score to discriminate patients into high versus low risk for needing pyeloplasty and compare these against models trained with single clinic visit data. We included patients followed for hydronephrosis from three institutions. The outcome of interest was low risk versus high risk of obstructive hydronephrosis requiring pyeloplasty. The model was trained on data from Toronto, ON and validated on an internal holdout set, and tested on an internal prospective set and two external institutions. We developed models trained with single ultrasound (single-visit) and multi-visit models using average prediction, convolutional pooling, long-short term memory and temporal shift models. We compared model performance by area under the receiver-operator-characteristic (AUROC) and area under the precision-recall-curve (AUPRC). A total of 794 patients were included (603 SickKids, 102 Stanford, and 89 CHOP) with a pyeloplasty rate of 12%, 5%, and 67%, respectively. There was no significant difference in developing single-visit US models using the first ultrasound vs. the latest ultrasound. Comparing single-visit vs. multi-visit models, all multi-visit models fail to produce AUROC or AUPRC significantly greater than single-visit models. We developed ML models for hydronephrosis that incorporate multi-visit inference across multiple institutions but did not demonstrate superiority over single-visit inference. These results imply that the single-visit models would be sufficient in aiding accurate risk stratification from single, early ultrasound images.

## Author summary

Pediatric hydronephrosis is a common imaging finding in children, and while the majority of patients do not require treatment, all patients undergo ultrasound monitoring through infancy to determine which will require surgery. In this study, we explored the potential of machine learning to predict need for surgery in children with pediatric hydronephrosis. Current models include data from a single ultrasound to predict this outcome. Using data from multiple pediatric hospitals, we developed models using single versus multiple ultrasounds from different visits with different fusion methods. We found that models trained with data from multiple visits were not superior to single-visit models. Interestingly, single-visit models demonstrated comparable model performance, regardless of whether they were trained on the first or latest ultrasound. These findings suggest that single-visit ultrasound models are sufficient for accurately predicting the risk of obstructive hydronephrosis, making them a practical and efficient option for clinical use. Our work highlights the robustness of single-visit models and informs future efforts to integrate machine learning into pediatric urology.

## Introduction

More than 80% of children with antenatal hydronephrosis (HN) do not require surgical intervention [1]. Differentiating between patients with surgical indications, such as ureteropelvic junction obstruction (UPJO), transient HN, congenital megaureter, vesicoureteral reflux, among other etiologies is an important clinical question. Usually, serial ultrasound and close clinical follow-up are performed from infancy to toddler age, with significant institutional variation in workup and management [2]. Among these, UPJO is the most common etiology warranting surgery with pyeloplasty. Early identification of at-risk patients requiring pyeloplasty is of significant clinical interest, and machine learning (ML) methods have recently been described to address this problem [3].

Current ML models in HN have been developed to predict the need for surgical intervention, the risk of re-intervention after surgery, and the necessity for invasive testing [4,5]. We have previously developed a deep learning model which predicted low versus high risk of pyeloplasty for isolated HN from two ultrasound images (sagittal and transverse views) of the affected kidney [6]. This model was more reliable than models employing supervised learning with clinical parameters to predict the need for pyeloplasty [3,7,8]. Namely, the hydronephrosis severity index was shown to reliably maintain high sensitivity for obstructive hydronephrosis while triaging low-risk patients and being assessed in silent trials [9]. However, children with HN often receive serial ultrasound imaging over multiple follow-ups and interval changes at each visit, which may be clinically useful in determining interventions such as additional testing, surgery or safe discharge from the clinic. This raises an important consideration for ML models, such as in Erdman (2020), on whether imaging data over follow-up is beneficial to predict the need for surgery or if data from the most recent visit is sufficient [6].

This study aims to determine whether incorporating ultrasounds over multiple follow-up visits improves the classification of infants with HN to determine low versus high risk of pyeloplasty to aid clinical decision-making. We applied ML methods from video-based modeling, namely, action recognition. This allows the temporal fusion of features used for the convolutional neural networks (CNN) applied in the original model [3,6]. We then compared the original single-visit and multi-visit models trained with various temporal fusion methods.

## Methods

### Ethics, study design, and problem

Each site received approval from their respective Internal Review Boards (IRB) and Research Ethics Boards (REB) for this work. Deidentified data was collected via retrospective chart review and therefore a waiver of consent was applied. Specifically, approval for data collection and analysis was granted by the Hospital for Sick Children REB (#1000053438) with appropriate data sharing agreements with the Children’s Hospital of Philadelphia IRB, and the Lucile Packard Children’s Hospital IRB. All research was performed in accordance with relevant guidelines and regulations. As data was fully anonymized, written informed consent was not required for the parent/guardian of each patient, in accordance with each REB.

This study was conducted and reported in accordance with the STREAM-URO framework, and additional details are provided in S1 Table. This is a supervised binary classification problem to predict low versus high risk for surgery (binary outcome), using ML models that are developed from (1) ultrasound images from a single clinic visit (baseline model) versus (2) multiple ultrasounds from consecutive visits (comparator models). In the original model, the hydronephrosis severity index is calculated as a continuous score from 0 to 1 to predict need for surgery, while in this study, it was binarized to simplify analysis.

### Data sources and splitting

Research ethics board approval was acquired for each included institution. Data was collected from the original model, trained at The Hospital for Sick Children (Toronto, ON) (2005–2021). External validation data was obtained from Lucile Packard Children’s Hospital (Stanford) (2018–2020) and Children’s Hospital of Philadelphia (CHOP) (1996–2020). HN patients who were seen in the paediatric urology clinic between 2015 and 2019 at SickKids, were less than 24 months of age at baseline, and had ultrasound findings of isolated hydronephrosis or hydroureteronephrosis (HUN), were included in this study. Patients with vesicoureteral reflux (VUR) or primary obstructive megaureter were included if those diagnoses were made after initial suspicion for UPJO-like hydronephrosis. Patients with VUR detected after a urinary tract infection (UTI) without evidence of HN, as well as those with known congenital anomalies of the urinary tract - such as duplication anomalies, posterior urethral valves and neurogenic bladder - were excluded.

The SickKids dataset included a retrospective cohort, further divided into a training and internal validation set using a 70:30 random split, and a prospective cohort from a silent trial [9]. The latter and the two external cohorts were used for model testing.

### Data processing

For each patient, two fixed-plane (transverse and sagittal) ultrasound images were taken for the affected kidney, as previously described [6]. Each kidney is considered independent from one another, and in patients with bilateral disease, the more affected kidney is used for analysis. Ultrasounds taken over multiple visits comprise two ultrasound views for the affected kidney. Images were preprocessed as in the original study [6]. Center cropping was done to remove text annotations and ultrasound beam borders. Contrast limited adaptive histogram equalization was used to normalize the contrast of the images. Lastly, images were resized to 256x256 pixels. No other data augmentation techniques were performed, and no missing data was found.

### Single-visit baseline model development

The model from the original paper was used as the baseline [3]. It is a Siamese convolutional neural network that takes in 2 images from kidney ultrasound (transverse and sagittal plane) to predict the likelihood of needing surgery. It consists of 7 convolutional layers and 3 fully-connected layers. The two ultrasound images are each fed into seven convolutional layers and one linear layer; the outputs are then concatenated and passed through two more linear layers and a softmax for binary classification.

The baseline model was training using the described training split. Details regarding hyperparameter tuning are outlined in the S1 Text. As in the original paper, ultrasounds from a patient’s hospital visits were considered independent during training. We trained and compared two baseline models using the first or latest ultrasound. When compared against the multi-visit models, the baseline model used the latest ultrasound.

### Multi-visit model development

We wanted to determine if ultrasounds over multiple hospital visits contained spatio-temporal information that could improve the prediction of obstructive HN. We explored the following methods to adapt the baseline model to handle multi-visit inference, and details are provided in S1 Text. (1) Average prediction, a naive extension to the single-visit model, in which prediction is performed for each available time point and predictions are averaged over time. (2) Convolutional pooling, where aggregate convolutional image features are pooled across time, and a max operation is done to create weighting of image features to predict an outcome. (3) The temporal shift module is a newly proposed method which assesses temporal shifts on feature maps between convolutional layers. (4) Long short-term memory (LSTM) is a recurrent neural network combined with CNNs, where outputs from CNN are fed into the LSTM network to model temporal dependencies across multiple frames to capture the temporal evolution of features.

### Model evaluation

Area under the receiver operating characteristic (AUROC) and area under the precision-recall curve (AUPRC) were used to compare the performance of various models on the binary classification task. AUROC is a measure of discriminative performance commonly used in predictive modeling, whereby 0.5 represents random guessing while 1.0 represents perfect discrimination. AUPRC describes how well the model identifies positive patients without false positives. 95% confidence intervals were determined for each metric using bias-corrected and accelerated bootstrap. The analysis code, including all required packages, is available at https://github.com/stan-hua/temporal_hydronephrosis.

## Results

### Population and datasets

A total of 401 patient trajectories were available from SickKids (753 clinic visits total) and were split randomly 70/30 into a training dataset (321 patients, 600 clinic visits) and an internal validation dataset (80 patients, 153 clinic visits). The model evaluation included three additional validation datasets: a prospective dataset from SickKids (202 patients, 244 examples; 12% pyeloplasty rate), Stanford (102 patients, 204 examples; 5% pyeloplasty rate), and CHOP (89 patients, 89 examples; 67% pyeloplasty rate).

### Performance of baseline model using the first versus latest ultrasound

**Fig 1:**
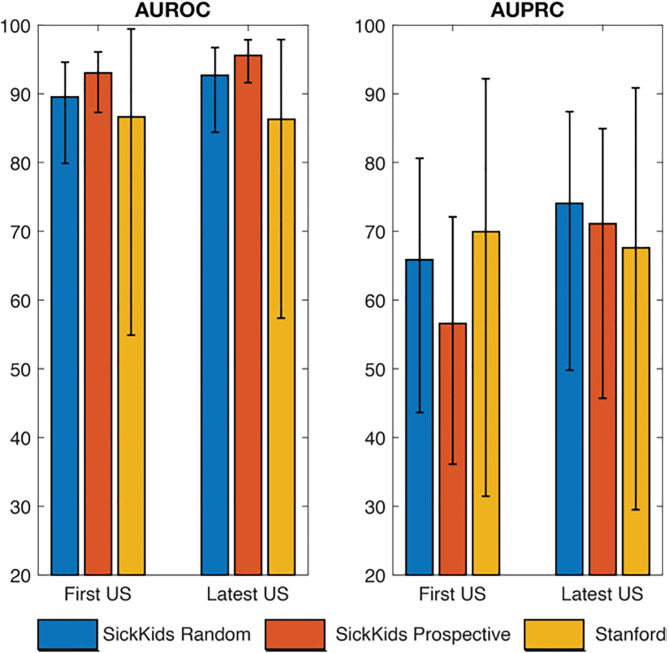
AUROC and AUPRC for baseline model trained to predict low versus high risk hydronephrosis using a single ultrasound (US) image at the first or latest clinic visit.

We first assessed if the performance varied between single-visit models trained with either first versus the latest ultrasound. The CHOP cohort was excluded as it included only single-visit examples. The AUROC and AUPRC are provided in Fig 1 (S2 Table). Evaluation across validation and testing datasets demonstrate no significant difference between first versus latest visit ultrasounds.

### Performance of single-visit versus multi-visit models

We then compared a single (baseline model trained on the latest ultrasound) model to a multi-visit model trained with average prediction, convolutional pooling, LSTM, and TSM for each testing set. We also include pre-trained models (using weights from the baseline model) for average prediction, convolutional pooling, and TSM models, as they only require modification of the forward pass of the single-visit model. This is contrary to retrained models, which did not use the previously determined weights in the single-visit model.

**Fig 2:**
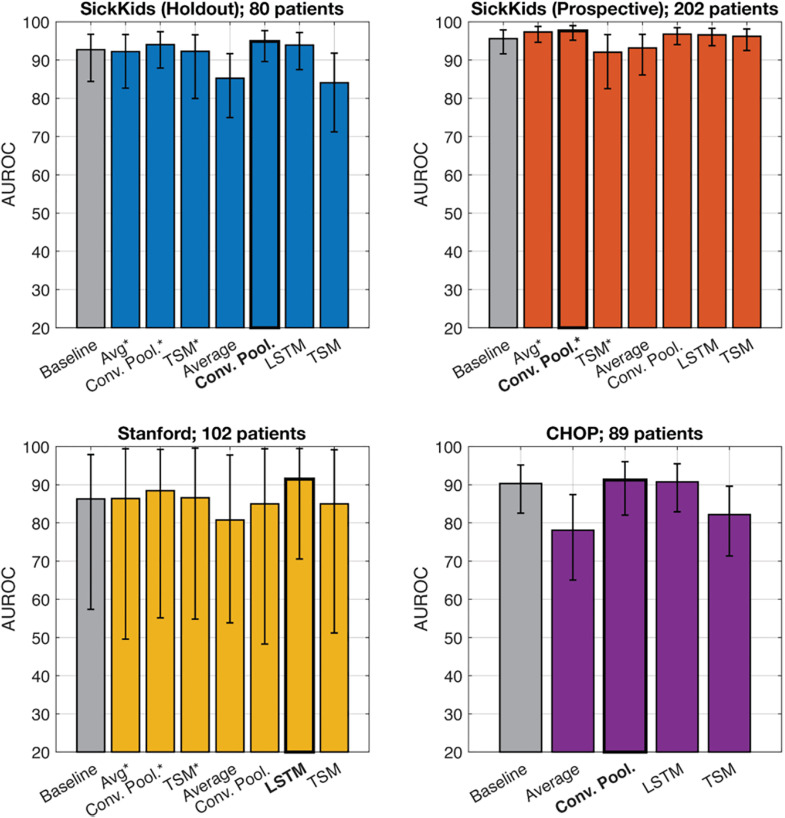
AUROC of single-visit (baseline using latest ultrasounds) versus multi-visit models trained with average prediction (Avg.), convolutional pooling (Conv. Pool), temporal shift model (TSM), and long short-term memory (LSTM). The asterisk signifies a pre-trained model. Model with highest AUROC bolded.

As shown in Fig 2 (S3 Table), all multi-visit models fail to produce AUROC or AUPRC values that are significantly better than single-visit models. While statistically not significant, multi-visit models trained with average prediction or TSM performed worse than baseline. Additionally, training multi-visit models with conv. pooling or LSTM does not impact single-visit inference, as seen in the performance on CHOP, which contains examples with only 1 visit each.

## Discussion

Here, we adapt the previously developed single-visit model to perform multi-visit inference through temporal fusion methods (average prediction, convolutional pooling, LSTM, TSM) to determine if it provides a benefit over single-visit models. We find that multi-visit models fail to perform better than the original single-visit model over each tested temporal fusion method. Additionally, we show no statistically significant difference in the prediction of low versus high risk hydronephrosis using the first or latest ultrasound. Together, this suggests that single-visit models may be sufficient for ML prediction in pediatric HN.

This work stems from an important question: in a clinical problem requiring multiple follow-up visits to reach a diagnosis, does adding multi-visit data improve model prediction? Currently, there are 8 published ML models in HN, all trained on single-visit information [5,10]. Most models are trained with clinical, ultrasound, and diuretic renogram data using supervised methods [7,11,12]. To date, only one model incorporates temporal data within its prediction, and an interval decrease in renal pelvis diameter was associated with a lower risk of re-intervention [13]. However, this model cannot incorporate unlimited multi-visit information; it inputs the most recent versus initial ultrasound. In contrast, models built with action recognition models can incorporate unlimited visits within the prediction.

The work should be considered considering several limitations. First, the external validation datasets from Stanford and CHOP were small, and the latter only included observations from a single clinical visit yet trained as a multi-visit model. Additionally, the pyeloplasty rates varied significantly between institutions which suggests potential selection bias and variable indications for surgery across centers. For this reason, the findings from this study should not extend beyond our institution but do share important insights on ML principles. Next, our model predicts obstruction by the decision to perform pyeloplasty. While our indications for surgery are aligned with current best practices, this incurs a degree of subjectivity to the outcome, although still clinically meaningful to patients. Next, the dataset was developed on patients who were initially presenting with UPJO-like hydronephrosis, and this resulted in the inclusion of a minority of patients who were later diagnosed with VUR or megaureter, which limits the internal validity of the model. Lastly, this data comes from a retrospective review of patients undergoing imaging at each institution and may be prone to selection bias. Some patients had fewer ultrasounds over their clinical course and the difference between multi-visit and single-visit models may be reduced in these selected patients. Given the slight performance benefits noted in this work, future work will expand on convolutional pooling models with additional multi-visit data. Altogether, our work shows that in cases where multi-visit data and models are not available, this is not a significant limitation hindering the development of clinically meaningful models. While promising, the results of this model should not be used to prematurely discharge or indicate surgery independent of clinical expertise.

## Conclusion

This multi-institutional study found no significant difference in single-visit prediction using ultrasounds from a patient’s first or last hospital visit. In addition, incorporating ultrasounds from a patient’s previous hospital visits does not significantly improve the prediction of renal obstruction over using just the most recent ultrasound. In a clinical setting, these results imply that the single-visit model may be sufficient to diagnose obstructive hydronephrosis, given kidney ultrasounds from any of the patient’s hospital visits.

## Supporting information

S1 Text Supplementary Methods. Hyperparameter Tuning. Average Prediction. Convolutional Pooling. Temporal Shift Module and Long short-term memory
(DOCX)

S1 Table STREAM-URO Reporting Checkbox. (DOCX)

S2 Table Single-visit baseline model’s performance given ultrasounds from a patient’s first hospital visit versus their latest hospital visit. 95\% bootstrapped confidence intervals are provided in brackets.
(DOCX)

S3 Table Comparison of model performance of single-visit versus multi-visit models. “(Pretrained)” denote using weights of pretrained baseline model with modification to perform multi-visit inference. As CHOP data is cross-sectional, adapted pretrained single-visit models are not included since these models default to the single-visit model when predicting on single-visit patients. 95% bootstrapped confidence intervals are provided in brackets. Top models according to AUROC and AUPRC for each test set are indicated with bold typeface.
(DOCX)
