# Supplementary material for: Longitudinal image-based prediction of surgical intervention in infants with hydronephrosis using deep learning: Is a single ultrasound enough?
Source: PLOS Digit Health. 2025 Aug 4;4(8):e0000939. doi: 10.1371/journal.pdig.0000939 (PMC12321052; doi:10.1371/journal.pdig.0000939)
Supplement: S1 Text — Hyperparameter Tuning. Average Prediction. Convolutional Pooling. Temporal Shift Module and Long short-term memory (DOCX) [file pdig.0000939.s001.docx]

**S1 Text: Supplementary Methods**

*Hyperparameter Tuning*

A randomized grid search with 5-fold cross-validation on the training set was performed to find optimal hyperparameters for the baseline model. Hyperparameters with the best average validation AUPRC across folds were kept. Negative log-likelihood loss was optimized via Stochastic Gradient Descent (SGD) with a learning rate of 0.005, momentum of 0.9, weight decay of 0.0005, and a batch size of 16.

For the multi-visit models, as the number of hospital visits varies with each example, this corresponds to a varying image-sequence length. Each example was fed into the model one at a time during training, validation and testing. Gradients were accumulated over examples to test for different effective batch sizes. Similar to the baseline, a randomized grid search for hyperparameters was done when training models with each of the following methods. Models were implemented in *pytorch-lightning* and trained on an NVIDIA RTX 3080 GPU.

*Average Prediction*

(Avg. Pred) is a naive extension to the single-visit baseline model, in which prediction is performed for each available time point, and the predictions are averaged over time. The single-visit model predicts ultrasounds from each hospital visit, and the mean is taken over the logit outputs. The mean logits are passed into a softmax as in the single-visit model.

*Convolutional Pooling*

(Conv. Pooling) has been effective in video classification tasks, performing significantly better than single-frame models and early 3D convolutional neural networks (CNN) [(Yue-Hei Ng et al. 2015; Tran et al. 2015)](https://paperpile.com/c/bO5XFU/VlUQP+6dkQm). It is a pooling method to aggregate convolutional image features across time, where a max operation is performed on features extracted from the last convolutional layer. In the adapted forward pass, the 1024-dimensional feature vectors (from concatenating the outputs of the Siamese layers) are extracted for each time point. A max operation is done on extracted features over time, and the resulting 1024-dimensional feature vector is passed through the remaining layers.

*Temporal Shift Module and Long short-term memory*

(TSM) is a more recently proposed method for temporal fusion that showed great performance on action recognition benchmarks [(Lin, Gan, and Han 2019)](https://paperpile.com/c/bO5XFU/FPs2u). Built on top of 2D CNNs, TSM allows the modeling of temporal dynamics through “temporal shifts” on feature maps between convolutional layers.

Between convolutional layers, the previous layer's output is a feature map of the form (T, C, H, W), where T is the number of time points, C is the number of channels, H is the height and W is the width. A portion of the feature map’s channels (C) is shifted once forward across time, and a non-overlapping portion is shifted once backwards across time. Adapting the single-visit model, temporal shifts occur between the Siamese convolutional layers, which means each ultrasound view (sagittal and transverse) is shifted independently across time. After the convolutional layers, features from the latest time point are fed into the remaining layers of the model to produce a single prediction
