# Supplementary material for: Longitudinal image-based prediction of surgical intervention in infants with hydronephrosis using deep learning: Is a single ultrasound enough?
Source: PLOS Digit Health. 2025 Aug 4;4(8):e0000939. doi: 10.1371/journal.pdig.0000939 (PMC12321052; doi:10.1371/journal.pdig.0000939)
Supplement: S1 Table — (DOCX) [file pdig.0000939.s002.docx]

**S1 Table:** STREAM-URO Reporting Checkbox

| **Methods** | |
| --- | --- |
| Problem | Supervised learning, binary classification |
| Source of data | 1. Hospital for Sick Children, Ontario, Canada 2. Lucile Packard Children’s Hospital, California, USA 3. Children’s Hospital of Philadelphia, Pennsylvania, USA |
| Eligibility Criteria | Children with hydronephrosis with available ultrasound visits including sagittal and transverse planes, multiple clinic visits with multiple images |
| Label | Low versus High Risk hydronephrosis (based on hydronephrosis severity index, with high risk indicating > 50% risk of surgery) |
| Data abstraction, cleaning, preparation | Single images were taken of sagittal and transverse views of the affected kidney. Thus, two images were taken per clinic visit. Center cropping was done to remove text or identifiable information. The images were resized to 256 x 256 pixels.  Ultrasound images from only one visit are available at CHOP, thus CHOP data is treated as sequences of 1. Per-patient multi-visit data of variable length were collected from SickKids and Lucile Packard Children’s and are coded with the number of visits specific to the patient. |
| Data splitting | Training: The Hospital for Sick Children (SickKids), Internal Training/Holdout (70:30)  Internal Testing: SickKids, Prospective Validation Cohort  External Validation Datasets: Lucile Packard Children’s Hospital (Stanford), Children’s Hospital of Philadelphia (CHOP). |
| Reference standard | NA |
| Model selection | Convolutional Neural Network (CNN), Siamese 2D architecture   - Average Prediction - Convolutional pooling - Temporal Shift Modules - Long short-term memory (RNN) |
| Hyperparameter tuning | - A randomized grid search with 5-fold cross-validation on training dataset for hyperparameters was done for training models of baseline model - Hyperparameters with the best average validation AUPRC across folds were kept. - Negative log-likelihood loss was optimized via Stochastic Gradient Descent (SGD) with a learning rate of 0.005, momentum of 0.9, weight decay of 0.0005, and a batch size of 16 |
