# Supplementary material for: Longitudinal image-based prediction of surgical intervention in infants with hydronephrosis using deep learning: Is a single ultrasound enough?
Source: PLOS Digit Health. 2025 Aug 4;4(8):e0000939. doi: 10.1371/journal.pdig.0000939 (PMC12321052; doi:10.1371/journal.pdig.0000939)
Supplement: S2 Table — 95\% bootstrapped confidence intervals are provided in brackets. (DOCX) [file pdig.0000939.s003.docx]

**S2 Table.** Single-visit baseline model's performance given ultrasounds from a patient's first hospital visit versus their latest hospital visit. 95\% bootstrapped confidence intervals are provided in brackets.

| **Dataset** | **Model** | **AUROC (95% CI)** | **AUPRC (95% CI)** |
| --- | --- | --- | --- |
| **SickKids Random Test** | **First** | 90 (80, 95) | 66 (44, 81) |
|  | **Latest** | 93 (84, 97) | 74 (50, 87) |
| **SickKids Prospective Test** | **First** | 93 (87, 96) | 57 (36, 72) |
|  | **Latest** | 96 (92, 98) | 71 (46, 85) |
| **Lucile Packard Children’s Hospital** | **First** | 87 (55, 99) | 70 (31, 92) |
|  | **Latest** | 86 (57, 98) | 68 (29, 91) |
