# Supplementary material for: Longitudinal image-based prediction of surgical intervention in infants with hydronephrosis using deep learning: Is a single ultrasound enough?
Source: PLOS Digit Health. 2025 Aug 4;4(8):e0000939. doi: 10.1371/journal.pdig.0000939 (PMC12321052; doi:10.1371/journal.pdig.0000939)
Supplement: S3 Table — “(Pretrained)” denote using weights of pretrained baseline model with modification to perform multi-visit inference. As CHOP data is cross-sectional, adapted pretrained single-visit models are not included since these models default to the single-visit model when predicting on single-visit patients. 95% bootstrapped confidence intervals are provided in brackets. Top models according to AUROC and AUPRC for each test set are indicated with bold typeface. (DOCX) [file pdig.0000939.s004.docx]

**S3 Table.** Comparison of model performance of single-visit versus multi-visit models. “(Pretrained)” denote using weights of pretrained baseline model with modification to perform multi-visit inference. As CHOP data is cross-sectional, adapted pretrained single-visit models are not included since these models default to the single-visit model when predicting on single-visit patients. 95% bootstrapped confidence intervals are provided in brackets. Top models according to AUROC and AUPRC for each test set are indicated with bold typeface

| Dataset | Model | AUROC (95% CI) | AUPRC (95% CI) |
| --- | --- | --- | --- |
| SickKids Random Test | Baseline | 93  (84, 97) | 74  (50, 87) |
|  | Average (Pretrained) | 92  (83, 97) | 75  (50, 87) |
|  | Convolutional Pooling (Pretrained) | 94  (88, 97) | 76  (52, 88) |
|  | Temporal Smoothing (Pretrained) | 92.26  (80, 97) | 76  (55, 88) |
|  | Average | 85  (75, 92) | 59  (38, 75) |
|  | Convolutional Pooling | **95**  **(90, 98)** | **79**  **(58, 90)** |
|  | LSTM | 94  (87, 97) | 79  (59, 90) |
|  | Temporal Smoothing | 84  (71, 92) | 68  (47, 82) |
| SickKids Prospective Test | Baseline | 96  (92, 98) | 71  (46, 85) |
|  | Average (Pretrained) | 97  (95, 99) | 73  (49, 87) |
|  | Convolutional Pooling (Pretrained) | **98**  **(95, 99)** | 74  (50, 88) |
|  | Temporal Smoothing (Pretrained) | 92  (83, 97) | 71  (48, 84) |
|  | Average | 93  (86, 97) | 71  (48, 84) |
|  | Convolutional Pooling | 97  (94, 99) | **77**  **(56, 89)** |
|  | LSTM | 97  (94, 98) | 68  (47, 83) |
|  | Temporal Smoothing | 96  (92, 98) | 74  (54, 87) |
| Stanford | Baseline | 86  (57, 98) | 68  (29, 91) |
|  | Average (Pretrained) | 86  (50, 99) | **73**  **(36, 94)** |
|  | Convolutional Pooling (Pretrained) | 88  (55, 99) | 70  (32, 92) |
|  | Temporal Smoothing (Pretrained) | 87  (55, 100) | 74  (36, 95) |
|  | Average | 81  (54, 98) | 58  (20, 85) |
|  | Convolutional Pooling | 85  (48, 99) | 71  (32, 93) |
|  | LSTM | **91**  **(71, 99)** | 71  (32, 93) |
|  | Temporal Smoothing | 85  (51, 99) | 61  (23, 88) |
| CHOP | Baseline | 90  (83, 95) | 95  (90, 98) |
|  | Average | 78  (65, 87) | 84  (69, 92) |
|  | Convolutional Pooling | **91**  **(82, 96)** | 95  (88, 92) |
|  | LSTM | 91  (83, 96) | **96**  **(92, 98)** |
|  | Temporal Smoothing | 82  (71, 90) | 91  (80, 95) |
